# The association of sleep problem, dietary habits and physical activity with weight status of adolescents in Nepal

**DOI:** 10.1186/s12889-021-10985-5

**Published:** 2021-05-17

**Authors:** Devendra Raj Singh, Dev Ram Sunuwar, Babita Dahal, Rajeeb Kumar Sah

**Affiliations:** 1 Department of Public Health, Asian College for Advance Studies, Purbanchal University, Lalitpur, Nepal; 2 Southeast Asia Development Actions Network (SADAN), Lalitpur, Nepal; 3 Department of Nutrition and Dietetics, Nepal Armed Police Force Hospital, Kathmandu, Nepal; 4 Department of Nursing, Asian College for Advance Studies, Purbanchal University, Lalitpur, Nepal; 5 School of Allied and Public Health Professions, Canterbury Christ Church University, Canterbury, Kent UK

**Keywords:** Sleep problems, Dietary habits, Physical activity, Weight status, Adolescents, Nepal

## Abstract

**Background:**

Overweight/obesity among adolescents is an emerging public health issue worldwide. However, the evidence on the determinants of body weight status and lifestyle behaviors among Nepalese adolescents is limited. This study aims to explore the sleep characteristics, dietary habits, and physical activity and its association with body mass index (BMI) among Nepalese adolescents.

**Methods:**

A cross-sectional study was conducted between July and November 2019 among 627 randomly selected adolescents from eight schools located in Kathmandu Metropolitan City, Nepal. A self-administrated structure questionnaire was used to collect the data. Anthropometric measurements (adolescent’s BMI), sleep characteristics, dietary habits, and physical activity were assessed using validated tools. Multinomial logistic regression analyses assessed the association between covariates and BMI categories. The statistical significance was considered at *p*-value < 0.05 and 95% confidence intervals (CIs).

**Results:**

The overall prevalence of underweight and overweight/obesity among adolescents was 9.1% (95% CI: 7.1–11.6) and 23.7% (95% CI: 20.6–27.7) respectively. In multinomial logistic regression, adolescents who reported sleep problem compared to those with no such problem (Relative risk ratio (RRR) = 13.37, 95% CI: 7.14–25.05), adolescents who had obstructive sleep apnea (OSA) symptoms (RRR = 3.21, 95% CI:1.31–7.86), who consumed soft drink ≥1 time/day in past 1 months (RRR = 5.44, 95% CI: 2.93–10.10), consumed high-fat dietary ≥2 times/day (RRR = 2.17, 95% CI: 1.18–3.99), and had a habit of junk food consumptions (RRR = 5.71, 95% CI:2.55–12.82), adolescents who had 5–6 h/day sedentary behavior (RRR = 3.21, 95% CI: 1.14–9.09), adolescents from Terai/Madhesi castes (RRR = 2.81, 95% CI: 1.19–6.64) and adolescents whose father was employed (RRR = 2.04, 95% CI: 1.04–3.98) were at increased risk of being overweight/obesity. In contrast, adolescents aged 14–16 years had 71% lower (RRR = 0.29, 95% CI: 0.16–0.52), and adolescents who consumed less than five food groups had 45% lower (RRR = 0.55, 95% CI: 0.31–0.97) risk of being overweight/obesity compared to 12–14 years age groups and consumed more than five food groups respectively.

**Conclusions:**

The findings of this study warrant immediate interventions to improve the lifestyle to reduce overweight/obesity among Nepalese adolescents. Creating a conducive environment, both at school and home is essential to encourage adolescents for the adoption of healthy lifestyle behaviors.

## Background

Overweight and obesity among adolescents aged 10–19 years are emerging public health issues worldwide [1, 2]. The global prevalence of overweight and obesity among children and adolescents, aged 5–19 years, has increased rapidly from 4% in 1975 to 18% in 2016 [3, 4]. Globally, it is estimated that about 158 million children aged 5–19 years are living with obesity in the year 2020 and projected to be 254 million by 2030 [5]. The prevalence is highest among the developed countries but there are increasing trends of overweight and obesity among children and adolescents in urban areas of the developing countries [3, 6, 7]. South and Southeast Asian countries, such as Nepal, already faced the burden of underweight and now the burden of overweight and obesity are increasing among children and adolescents, especially in the city areas of the country [3, 8, 9]. As a result, developing countries like Nepal are faced with the double burden of malnutrition, which is becoming a major public health concern [9, 10]. In Nepal, a low-income country located in the lap of the Himalayas between China and India, the prevalence of childhood overweight and obesity range from 14 to 19% and 7–11%, respectively [11, 12]. On the other hand, according to a recent global school-based student health survey report, about 11% of school-going Nepalese adolescents were underweight [13].

Adolescence is a critical period of transition from childhood to adulthood, and therefore, both individual and environmental factors influence their health behaviors, dietary choices, and social activities [14]. The overweight and obesity among adolescents are multifactorial conditions that are often linked to different lifestyle factors such as a change in sleep patterns, dietary habits, physical inactivity, mode of transportation, and sedentary lifestyle [15, 16]. The multiple influencing factors of overweight/obesity among adolescents can be broadly categorized into intrapersonal, interpersonal, organizational, and community-level factors [17, 18]. Due to the lack of complete psychosocial maturity among adolescents, they are assumed to be more vulnerable to adopt unhealthy behaviors that may have long-term adverse health and nutritional consequences [7]. Moreover, overweight and obese adolescents are likely to maintain their lifestyle choices into adulthood and later life, exposing themselves to the higher risks of several non-communicable diseases such as diabetes, cardiovascular diseases, and cancer [19]. Lifestyle choices such as adequate sleep, unhealthy dietary habits, physical inactivity, and sedentary lifestyles are key factors that can contribute to overweight and obesity among children and adolescents [20].

Adequate sleep is a biological necessity of human life and a critical cycle of our daily life that helps to sustain the normal function of the brain and maintain homeostasis [21, 22]. Good quality and adequate amount of sleep during adolescence are even more important for the proper maturation of the brain and good health [23]. According to the guidelines of the National Sleep Foundation, adolescents require a minimum of 8–10 h of sleep every day [24]. However modern lifestyle behaviors have contributed towards a decrease in the duration of sleeping hours among individuals and, sleeping disorders are becoming one of the emerging problems worldwide [25]. Previous studies suggest adolescents population does not get adequate sleep during school days which may put them at greater risk of long-term health problems [26, 27]. Also, short sleeping hours among adolescents is associated with increased body mass index (BMI), and reduced academic performance, and poor physical and mental health [23, 28, 29]. Similarly, unhealthy dietary habits and physical inactivity are key modifiable risk factors that largely contribute to the increase in overweight and obesity among adolescents [30]. Previous studies have suggested that the shift of dietary habits from traditional staple locally available foods towards consumptions of ultra-processed, energy-dense, high fat, and low fiber foods plays a crucial role in increasing the prevalence of overweight and obesity globally [8, 31].

Although lifestyle factors have been considered as key determinants for overweight and obesity, such findings in the previous studies are mostly presented from developed countries [32]. In most of the resource-poor countries, such as Nepal, the impact of nutrition transition has been observed [33, 34], however very little evidence has been generated on how lifestyle factors have been influencing adolescent’s weight status and how it differs when compared with evidence from western countries. Although the evidence from developed countries may be true for Nepalese children and adolescents, there is a deficient of such evidence within the Nepalese context.

In the last few decades, Nepal has undergone a massive demographic shift in population, where it is expected that in 2021, adolescents will comprise of about 19% of the total Nepalese population [35] in Nepal. Rapid urbanization and technological advancement in current years have presented adolescents with the freedom to modify their lifestyle in terms of sleep patterns, physical activity, and dietary behavior modifications [36]. A recent survey from Nepal indicated that the majority of adolescents consumed insufficient fruits and vegetables and were physically inactive [37]. Moreover, Nepal’s poor health-promoting policies in the areas of health, environment, education, dietary system, and agriculture are believed to reinforce adolescents in adopting risky health behaviors, including harmful lifestyle and unhealthy dietary choices among adolescents [36]. Although, previous studies have reported the increasing prevalence of adolescents overweight and obesity in urban settings in Nepal, the influences of sleep patterns, physical activity, and dietary habits on Nepalese adolescents’ weight status remained inadequately explored [11, 13]. This study provides evidence for a deeper understanding of how overweight and obesity among Nepalese urban adolescents differ with ethnic, environmental, economic, and behavioral factors. To the best of our knowledge, so far no studies have studied sleep problems as risk factors for overweight and obesity among adolescents in the Nepalese context. Therefore, this study aims to study sleep behaviors, dietary habits, and physical activity and its association with body mass index among Nepalese adolescents in the Kathmandu Metropolitan City.

## Methods

### Study setting and design

A cross-sectional study was conducted between July and November 2019, among adolescents aged 11–19 years in Kathmandu a Metropolitan City and capital of Nepal. Kathmandu Metropolitan City is a residence to 20% of Nepal’s urban population from a diverse ethnic and socio-cultural background and has a density of 13,225 population per square kilometers [38]. The city was selected purposively as the study site due to its cosmopolitan nature, which can better represent the Nepalese urban adolescents’ population.

### Sample size determination and sampling procedure

The sample size was calculated using the single population proportion formula: *N* = Z^2^pq/d^2^, considering 95% confidence interval (CI), 50% assumed proportion (p) [39], and 5% margin of error (d). Assuming a 10% non-response rate and 1.5 design effect, the sample size was estimated to be 635.

A multistage cluster random sampling method was used to select the study sample. In the first stage, all 32 local administrative units (locally known as a “Ward”) of Kathmandu Metropolitan City were considered as clusters for this study. There were a total of 91 public and 638 private schools within the Kathmandu Metropolitan City, with approximately a total of 165,000 adolescents; more than 90% of adolescents pursuing their education in private schools within the Kathmandu Metropolitan City [40]. Considering the cluster size, the number of students per class, geographical coverage, and time frame of the study, two clusters out of 32 clusters were randomly selected for the study sites. In the second stage, the total number of schools within the selected clusters were categorized into two separate categories of public and private schools. Then, from the two randomly selected clusters, an equal number of schools i.e. four private schools and four public schools from each category were again randomly selected for this study. Finally, within each randomly selected eight schools (four private schools and four public schools), two-classes per year group (from grade 8–10) were randomly selected. Subsequently, all adolescents’ (10–19 years) students from the selected classes were invited to fill up the questionnaires. Adolescents with serious health problems, including physical or psychological difficulties, or those who were absent on the days of data collection were excluded from the study.

### Data collection and study variables

Self-administrated questionnaires were distributed to a total of 635 eligible adolescents. The instructions about questionnaires were explained to the adolescents jointly by core study team members and health teachers of the respective schools before the adolescents filled up the questionnaires. Also, we collected the student’s information on the socio-demographic part from their parents by sending a questionnaire along with the consent form. The socio-demographic questionnaires completed by parents were collected at the respective schools of their children within the 4 days of the questionnaire distribution. Anthropometric measurements of children were taken at their respective schools. All tools were originally developed in English and then translated into Nepali language and back-translated to the English language to ensure that the originality of the questionnaire remains unchanged. Also, the tools were pretested among 50 adolescents from a school that was not included in the analytical samples. The data collectors were undergraduate nurses (BSc Nursing), and they were provided a four-day training. The training included an explanation of the objective of the study, data collection procedure, sampling method, ethical principles and guidelines, and data entry techniques. Approximately 20 min was required for adolescents to fill out the questionnaires.

### Outcomes variable

#### Weight status

Adolescent’s BMI was computed using the formula BMI = kg/m^2^ where kg was student’s weight in kilograms and m^2^ was their height in meters squared. The student’s height was measured to the nearest 0.1 cm using SECA ceo0123 portable Stadiometer. The participants were asked to take off their shoes and any object on the head before standing on the scale. Likewise, the weight of the participants with minimal clothes and without shoes was measured to the nearest 0.1 kg using calibrated digital bathroom weighing scales. The World Health Organization’s BMI-for-age guideline was used to classify the adolescents BMI scores into five categories: “very thin” (BMI for age between <5th percentile or < −3SD), “thin” (BMI for age between 5th -14th percentile or < − 2SD), “normal” (BMI for age between 15th -85th percentile or -2SD to +2SD), “overweight” (BMI for age between 85th -94th percentile or > +1SD to +2SD) and “obese” (BMI for the age ≥ 95th percentile or > +2SD) [41].

### Predictor variables

#### Sleep measures

Adolescents’ sleep habits and problems were assessed using the student version of the Child and Adolescents Sleep Checklist (CASC-s) [42], as this tool has been previously used in a similar cultural setting [43]. The tool is designed to assess sleep habits among preschoolers, and school (including high school) children. CASC-Student version consists of 24 questions related to sleep problems. The sleep disturbance score was calculated based on the responses of the 24 items questions recorded in a four-point Likert scale where 0 = never or unknown, 1 = occasionally (0 or 1 day per week), 2 = usually (2 to 4 days per week) and 3 = always (5 to 7 days per week). The total sum of score from 24 items sleeps disturbance questions range from 0 to 72. Children with CASC sleep disturbance score of 18 or more are considered to have a sleep problem. Also, the CASC score is grouped into four categories: “bedtime problem” (Q1 - Q6), sleep “breathing and unstable sleep” (Q7 - Q12), parasomnia, and sleep movement (Q13 - Q18), and “daytime problem” (Q19 - Q24). The bedtime and wake time during previous weekdays and weekends were obtained through a self-reported version of CASC where weekdays and weekends refer to the preceding week of data collection. Duration of sleep or total night-time sleep was computed by subtracting bedtime from wake time. Cronbach’s alpha of the tool in this study was 0.72.

Likewise, obstructive sleep apnea (OSA) symptoms, characterized by partial or complete obstruction of upper airways, were assessed by two-items related to individual snoring behavior and sleepiness feeling. Participants’ responses (as “true” or “partially true”) to the statement “I snore (or someone else says I snore)” and “feeling sleepiness at least three days per week” were classified as having OSA symptoms. This operational definition of obstructive sleep apnea symptoms has been previously used in other epidemiological studies [44, 45].

#### Dietary habits

Self-administrated Nepalese version of Global School-Based Adolescents Health Survey (GSHS) core questionnaire modules developed by the World Health Organization [46] was used to assess the dietary habits of adolescents during the last 30 days preceding the survey. The tools consist of nine items of questions related to the dietary habits of adolescents. The information was collected regarding the frequency of fruits consumption per day, frequency of vegetable consumed per day, frequency of foods high in fat (e.g., ghee, fried food, ice cream or cream doughnuts), frequency of salty foods consumption (e.g., instant noodles, potato crackers/chips, daalmoth, salty cookies, biscuits, fries or paapad) and frequency of consumptions of carbonated soft drinks (e.g., Coca-Cola, Pepsi, Mountain Dew, or sprite) per day in last 30 days. Likewise, the consumption of fast food, i.e., food from a restaurant (e.g., Mo: Mo, Chowmein, Burger, or Pizza) in the last 7 days. In addition, 24 h dietary recall method was also used to record food group consumptions (ten food groups) among adolescents in the last 24 h preceding the study [47]. Adolescents’ habits on meal patterns, vegetarianism, and junk food consumption were also recorded.

#### Physical activity and sedentary behavior

Physical activity questionnaires were adapted from the Nepalese version of Global School-Based Adolescents Health Survey (GSHS) core questionnaire modules developed by WHO [46]. The tool consists of five items of questions related to physical activity and also these tools have been used previously in a global school-based student survey in Nepal [48, 49]. Physical activity questionnaires in the GSHS survey module intended to collect information regarding physical activities of the individual student for the last 7 days, such as the total number of days per week he/she was physically active at least more than 60 min (running, fast walking, biking, dancing, football, volleyball, Kabaddi, and Cricket), the total number of days per week he/she walk or ride a bicycle to or from school/home, and the total number of days per week he/she go to physical education class. According to the WHO’s recommendation regarding physical activity for adolescents, an adolescent who accrues at least 60 min of moderate or vigorous-intensity physical activity per day is considered as physically active or in a good level of physical activeness [50]. Likewise, sedentary behavior in the last 7 days was assessed in terms of the number of hours spent on a typical day sitting, watching television, playing computer games, talking with friends, or doing other sitting activities.

#### Socio-demographic information

The socio-demographic information included: adolescents’ age, sex, grade, ethnicity (categorized as Brahmin/Chhetri, Janajati, Madheshi, Muslim, Dalit, and others based on ethnic categorization used by the government of Nepal in Nepal Demographic and Health Survey [51]), religion (Hindu versus non-Hindu), family type (nuclear and joint or extended), education level of father and mother (below high school: < grade 10, high school:10–12 grade, and university level), parents occupation (employed and unemployed), and family household income in Nepalese Rupees (< 10,000, 10,000-25,000, 25,001-50,000, and > 50,000). All socio-demographic variables were reported by the parents of adolescents.

### Statistical analysis

The collected data were entered into EpiData software 3.1v and transferred into StataMP version 14.1 (StataCorp LP, College Station, Texas) for statistical analyses. WHO Anthro Plus Software V.1.0.4 was used for BMI scores. The descriptive results are presented in the form of mean, standard deviation, frequency, and percentage. Both histogram and Kolmogorov-Smirnov normality test (*p* < 0.05) were applied to check the normal distribution of the data (z-score outliers). Pearson’s chi-square (χ2) and One-way analysis of variance (ANOVA) tests were applied to observe differences in socio-demographic characteristics, sleep measures, physical activity, and sedentary behaviors of participants by BMI categories. Multinomial logistic regression analyses were applied to observe the effects of the predictors on BMI categories considering “normal BMI” as a reference category. Multicollinearity amongst the predictor variables was checked using the Variance Inflation Factor (VIF). The mean value of VIF < 10 was the cut-off point [52]. The statistical significance was considered at *p*-value < 0.05 and 95% confidence intervals (CIs).

Four models were created to adjust the association between different predictors such as socio-demographic variables of parents and adolescents, sleep characteristics, dietary habits, and physical activity with the outcome of interest as BMI among adolescents using multinomial logistic regression analyses. Backward stepwise processes were used to identify parsimonious models to explain the predictors of adolescent’s weight status (all *p* < .05). Model-I was adjusted with the main predictor variables such as adolescents’ sleep characteristics and dietary habits. Model-II was adjusted for adolescents’ sleep characteristics, dietary habits, and socio-demographic characteristics of parents and adolescents. Model-III was adjusted with physical activity and socio-demographic characteristics of parents and adolescents. Model-IV adjusted for significant variables (*p* < 0.05) from Model- I, Model-II, and Model-III resulting in a parsimonious model in the multivariate analysis.

### Ethics

The ethical approval for this study was obtained from the ethical review board of the Nepal Health Research Council (Ref. no: 2777/2019). Permission was also taken from the participating schools. Written consent was obtained from the legal guardians or parents of eligible children aged 10–19 years. A separate questionnaire with an informed consent form and request letter from respective schools were sent to parents through their children. Written consent was also taken from the adolescents who participated in the study. Adolescents were also informed that they were allowed to leave any questions unanswered in the self-administered questionnaire. Participation was voluntary, and participants’ identity was kept confidential.

## Results

Table 1 depicts the socio-demographic information by weight status. Of the 635 adolescents who were provided a self-administered questionnaire, a total of 627 completed the questionnaire (response rate = 98.7%). The overall prevalence of underweight and overweight/obesity among adolescent students was 9.1% (95% CI: 7.1–11.6) and 23.7% (95% CI: 20.6–27.7) respectively.

**Table 1:** Socio-demographic related information by weight status of the study participants (*n* = 627)

Study variables	Total n (%)	Underweight n (%)	Normal n (%)	Overweight/obese n (%)	***P*** value^**1**^
Prevalence of BMI 95% CI	627 (100)	57 (9.1) [7.1–11.6]	421 (67.1) [63.4–70.7]	149 (23.7) [20.6–27.7]	
**Students characteristics**					
**Age (years) (mean ± SD)**	14.4 ± 1.1	15.1 ± 1.1	14.4 ± 1.1	13.9 ± 1.1	< 0.001*
**Age (years)**					
12–13	138 (22)	7 (12.2)	73 (17.3)	58 (38.9)	< 0.001*
14–16	489 (77.9)	50 (87.7)	348 (82.6)	91 (61.1)	
**Sex**					
Female	288 (45.9)	23 (40.3)	192 (45.6)	73 (48.9)	0.523
Male	339 (54.1)	34 (59.6)	229 (54.3)	76 (51)	
**Class/Grade**					
Eight	193 (30.7)	16 (28.1)	128 (30.4)	49 (32.8)	0.056
Nine	248 (39.5)	15 (26.3)	177 (42)	56 (37.5)	
Ten	186 (29.6)	26 (45.6)	116 (27.5)	44 (29.5)	
**Type of school**					
Public	196 (31.2)	26 (45.6)	133 (31.5)	37 (24.8)	0.015*
Private	112 (75.1)	31 (54.3)	288 (68.4)	112 (75.1)	
**Parents characteristics**					
**Ethnicity**					
Brahmin/Chhetri	225 (40.6)	14 (24.5)	174 (41.3)	67 (44.9)	< 0.001*
Terai Madhesi castes	68 (10.8)	4 (7)	42 (9.9)	22 (14.7)	
Dalit and minorities	89 (14.1)	25 (43.8)	50 (11.8)	14 (9.4)	
Janajati	215 (34.2)	14 (24.5)	155 (36.8)	46 (30.8)	
**Religion**					
Hindu	493 (78.6)	42 (73.6)	325 (77.2)	126 (84.5)	0.107
Non-Hindu	134 (21.3)	15 (26.3)	96 (22.8)	23 (15.4)	
**Family type**					
Nuclear	347 (55.3)	28 (49.1)	240 (57)	79 (53)	0.430
Joint	280 (44.6)	29 (50.8)	181 (42.9)	70 (46.9)	
**Mothers education**					
Below high school (≤10 class)	365 (58.2)	29 (50.8)	242 (57.4)	94 (63.1)	0.452
High class (10 + 2)	179 (28.5)	21 (36.8)	122 (28.9)	36 (24.1)	
University education	83 (13.2)	7 (12.2)	57 (13.5)	19 (12.7)	
**Fathers education**					
Below high school (≤ 10 class)	87 (13.8)	6 (10.5)	59 (14)	22 (14.7)	0.937
High class (10 + 2)	236 (37.6)	23 (40.3)	156 (37.1)	57 (38.2)	
University education	304 (48.4)	28 (49.1)	206 (48.9)	70 (46.9)	
**Mothers occupation**					
Unemployed	396 (63.1)	36 (63.1)	260 (61.7)	100 (67.1)	0.507
Employed	231 (36.8)	21 (36.8)	161 (38.2)	49 (32.8)	
**Fathers occupation**					
Unemployed	157 (25)	21 (36.8)	110 (26.1)	26 (17.4)	0.011*
Employed	470 (74.9)	36 (63.1)	311 (73.8)	123 (82.5)	
**Monthly family income (NRs)(1USD = 110NRs)**					
< 15,000	112 (17.8)	15 (26.3)	68 (16.1)	29 (19.4)	0.543
15,000–30,000	260 (41.4)	19 (33.3)	183 (43.4)	58 (38.9)	
30,000–45,000	132 (21.1)	13 (22.8)	87 (20.6)	32 (21.4)	
> 45,000	123 (19.6)	10 (17.5)	83 (19.7)	30 (20.1)	

^1^Denotes chi-square test and fisher exact test for categorical variables and one-way ANOVA for continuous variables

*Denotes statistically significant variables at *p* < 0.05

### Socio-demographic information of adolescents and parents

The mean (± SD) age of adolescents was 14.4 (± 1.1) years. More than three fourth (77.9%) of the adolescents were aged between 14 and 16 years, attended private schools (75.1%), and followed the Hindu religion (78.6%). More than half were male (54.1%), had a nuclear family type (55.3%). Slightly more adolescents were from grade nine. More parents were from the Brahmin/Chhetri ethnic group (40.6%), and more mothers attended below high school education (58.2%), while approximately half (48.4%) of fathers had attended university-level education. The majority of mothers were unemployed (63.1%), and three fourth of fathers were employed (74.9%), and more (41.4%) of parents had a family monthly income between 15,000–30,000 Nepalese Rupees (130–260 USD) per month (Table 1).

Underweight was most prevalent among 14–16 years (87.7%), males (54.1), private school attendant (54.3%), from Dalit and minority ethnicity (43.8%), and joint family (50.8%), whose mothers had less than high school education (50.8%) and unemployment (63.1%), unemployed fathers (63.1%), and having family monthly income between 15,000–30,000 NRs (33.3%) (Table 1).

Likewise, overweight/obesity was most prevalent among the age group 14–16 years (61.1%), males (51%), belonged to private school (75.1%), belonged to Brahmin/Chhetri ethnicity (44.9%), in a nuclear family (53%), amongst those whose mothers had below high school education (63.1%), in fathers university-level education (46.9%), in unemployment mothers (67.1%), in employed fathers (82.5%), and had family monthly income between 15,000–30,000 NRs (38.9%) (Table 1).

### Sleep characteristics of adolescents

The mean (± SD) sleep duration for weekdays and weekends was 8.9 (± 1.1) and 8 (± 1.1) hours, respectively. About one in four adolescents had sleep problems with a mean (± SD) CASC score of 24.7 (± 4.5), and one in ten had OSA symptoms (Table 2).

**Table 2:** Sleep duration, food habits, and physical activity related information by weight status of the study participants (*n* = 627)

Study variables	Total n (%)	Underweight n (%)	Normal n (%)	Overweight/obese n (%)	***P*** value^**1**^
**Sleep characteristics**					
**Sleep problem based on CASC Score**					
No sleep problem	474 (75.6)	49 (85.9)	360 (85.5)	65 (43.6)	< 0.001*
Sleep problem	153 (24.4)	8 (14)	61 (14.4)	84 (56.3)	
**No sleep problem, mean ± SD**	9.7 ± 3.8	10.1 ± 2.1	9.6 ± 3.7	10.3 ± 5.1	0.281
**Sleep problem, mean ± SD**	24.7 ± 4.5	21.0 ± 2.1	23.2 ± 3.5	26.2 ± 4.7	< 0.001*
**CASC score, mean ± SD**					
Total score	13.4 ± 7.6	11.6 ± 4.7	11.5 ± 6.1	19.3 ± 9.2	< 0.001*
Bed time problem	4.5 ± 2.9	3.9 ± 1.7	3.9 ± 2.4	6.6 ± 3.5	< 0.001*
Breathing and unstable sleep	3.6 ± 3.4	4.1 ± 3.3	2.6 ± 2.8	6.1 ± 3.1	< 0.001*
Parasomnia and sleep movement	2.2 ± 2.1	1.3 ± 1.5	2 ± 1.9	3.2 ± 2.3	< 0.001*
Day time problem	2.9 ± 1.9	2.5 ± 1.2	2.9 ± 1.9	3.2 ± 2.1	0.028*
**Obstructive sleep apnea (OSA)**					
No	570 (90.9)	53 (92.9)	398 (94.5)	119 (79.8)	< 0.001*
Yes	57 (9.1)	4 (7.1)	23 (5.4)	30 (20.1)	
**Sleep duration weekdays (hours), mean ± SD**	8.9 ± 1.1	8.7 ± 1.1)	9.1 ± 1	8.4 ± 1.2	< 0.001*
**Sleep duration weekends (hours), mean ± SD**	8 ± 1.1	8.3 ± 1.3	8.1 ± 1.1	7.6 ± 0.9	< 0.001*
**Adolescents food habit in past 30 days**					
**Went hungry**					
Never	383 (61.1)	28 (49.1)	264 (62.7)	91 (61.1)	0.142
Rarely/sometimes	244 (38.9)	29 (50.8)	157 (37.2)	58 (38.9)	
**Consumed fruits**					
Never	320 (51)	22 (38.6)	235 (55.8)	63 (42.2)	0.003*
≥ 1 times/day	307 (48.9)	35 (61.4)	186 (44.1)	86 (57.7)	
**Consumed vegetables**					
≤ 1 time/day	161 (25.6)	16 (28.1)	105 (24.9)	40 (26.8)	0.820
≥ 2 times/day	466 (74.3)	41 (71.9)	316 (75.1)	109 (73.1)	
**Consumed soft drinks**					
< 1 time/day	294 (46.8)	43 (75.4)	226 (53.6)	25 (16.7)	< 0.001*
≥ 1 time/day	333 (53.1)	14 (24.5)	195 (46.3)	124 (83.2)	
**Consumed high fat food**					
≤ 1 time/day	409 (65.2)	38 (66.6)	297 (70.5)	74 (49.6)	< 0.001*
≥ 2 times/day	218 (34.7)	19 (33.3)	124 (29.4)	75 (50.3)	
**Consumed fast food**					
Daily	65 (10.3)	15 (26.3)	38 (9)	12 (8.1)	< 0.001*
2–3 days/week	335 (53.4)	26 (45.6)	238 (56.5)	71 (47.6)	
≥ 4 days/week	227 (36.2)	16 (28.1)	145 (34.4)	66 (44.3)	
**Consumed junk food**					
No	185 (29.5)	13 (22.8)	156 (37.1)	16 (10.7)	< 0.001*
Yes	133 (89.2)	44 (77.1)	265 (62.9)	133 (89.2)	
**Frequency of consumed junk food**					
None	185 (29.5)	13 (22.8)	156 (37.1)	16 (10.7)	< 0.001*
1–3 times/week	289 (46.1)	35 (61.4)	195 (46.3)	59 (39.6)	
≥ 4 times/week	153 (24.4)	9 (15.7)	70 (16.6)	74 (49.6)	
**Consumed food groups (10 food groups)**					
< 5 food groups	277 (44.1)	39 (68.4)	168 (39.9)	70 (46.9)	< 0.001*
≥ 5 food groups	350 (55.8)	18 (31.5)	253 (60.1)	79 (53)	
**Adolescents physical activity**					
**Students physical activity**					
Not active	396 (63.1)	48 (84.2)	249 (59.1)	99 (66.4)	0.001*
Active	50 (33.5)	9 (15.7)	172 (40.8)	50 (33.5)	
**Walking or ride bicycle**					
None	325 (51.8)	36 (63.1)	227 (53.9)	62 (41.6)	0.014*
1–3 days	111 (17.7)	11 (19.3)	66 (15.6)	34 (22.8)	
≥ 4 days	191 (30.4)	10 (17.5)	128 (30.4)	53 (35.5)	
**Physical education**					
None	353 (56.3)	42 (73.6)	209 (49.6)	102 (68.4)	< 0.001*
1–3 days	113 (18)	5 (8.7)	79 (18.7)	29 (19.4)	
≥ 4 days	161 (25.6)	10 (17.5)	133 (31.5)	18 (12.1)	
**Sedentary behavior**					
< 1 h/day	80 (12.7)	12 (21.1)	51 (12.1)	17 (11.4)	< 0.001*
1–2 h/day	208 (33.1)	18 (31.5)	151 (35.8)	39 (26.1)	
3–4 h/day	267 (42.5)	23 (40.3)	192 (45.6)	52 (34.9)	
5–6 h/day	72 (11.4)	4 (7)	27 (6.4)	41 (27.5)	

^1^Denotes chi-square test and fisher exact test for categorical variables and one-way ANOVA for continuous variables

*Denotes statistically significant variables at *p* < 0.05

Underweight was most prevalent among adolescents without sleep problems (85.9%) and history of OSA symptoms (92.9%). Conversely, overweight/obesity was most prevalent among adolescents who had sleep problems (56.3%) but no history of OSA symptoms (79.8%) (Table 2). The mean score ± SD of bedtime problem (6.6 ± 3.5), breathing and unstable sleep (6.1 ± 3.1), parasomnia and sleep movement (3.2 ± 2.3), and day time problem (3.2 ± 2.1) among overweight/obese adolescents (Table 2).

### Dietary habits of adolescents

Only 38.9% of adolescents felt hungry due to not enough food in the home, half (51%) of adolescents never consumed fruits, one fourth (74.3%) of adolescents consumed vegetables more than two times a day, more (53.1%) of adolescents drank soft drinks, and one third (65.2%) of adolescents had consumed high-fat foods less than once a day in past 30 days. About nine in every ten children had consumed junk food, nearly half (46.1%) of adolescents have consumed junk food 1–3 times per week, more than half (53.4%) of adolescents took fast food 2–3 days per week, and more (55.8%) adolescents had consumed more than five food groups (Table 2).

Underweight was most prevalent among those adolescents who rarely/sometimes went hungry in school (50.8%), consumed fruits ≥1 times/day (61.4%), consumed vegetables ≥2 times/day (71.9%), consumed soft drinks < 1 time/day (75.4%), consumed high-fat food ≤1 time/day (66.6%), consumed fast food 2–3 days per week (45.6%), had consumed junk food (77.1%), consumed junk food 1–3 times per week (61.4%), and consumed less than 5 food groups (68.4%). Similarly, overweight/obesity was most prevalent among those adolescents who never went hungry in school (61.1%), consumed fruits ≥1 times/day (57.7%), consumed vegetables ≥2 times/day (73.1%), consumed soft drinks ≥1 time/day (83.22%), consumed high-fat food ≥2 time/day (50.3%), consumed fast food 2–3/days per week (47.6%), consumed junk food (89.2%), consumed junk food ≥4 times per week (49.6%), and consumed ≥5 food groups (53%) (Table 2).

### Physical activity and sedentary behaviors of adolescents

More adolescents (63.1%) were not physically active, and half of the adolescents (51.8%) didn’t walk or ride a bicycle in the past 7 days. The majority of the adolescents (56.3%) didn’t get any physical education/training during the past school year, and more adolescents (42.5%) spent 3–4 h per day in sedentary behaviors (Table 2).

Underweight was most prevalent among those adolescents who weren’t physically active (84.2%), didn’t walk or ride a bicycle (63.1%) in the past 7 days, who didn’t get any physical education during this school year (73.6%), and who had spent 3–4 h/day in sedentary behavior (40.5%). Likewise, overweight/obesity was most prevalent among those adolescents who weren’t physically active (66.4%), didn’t walk or ride a bicycle as their primary means of transport to and from school or around the home (41.6%) in the past 7 days, who didn’t get any physical education during this school year (68.4%), and who had spent 3–4 h/day in sedentary behavior (34.9%) (Table 2).

### Factors associated with weight status among adolescents

In this study, Table 3 shows relative risk ratios of adjusted multinomial logistic regression models for underweight and overweight/obese relative to normal-weight adolescents.

**Table 3:** Multivariate multinomial logistic regression analysis for weight status and its associated factors among adolescents (*n* = 627)

	Model-I		Model-II		Model-III		Model-IV	
Study variables	Underweight	Overweight/obese	Underweight	Overweight/obese	Underweight	Overweight/obese	Underweight	Overweight/obese
	RRR(95% CI)	RRR(95% CI)	RRR(95% CI)	RRR(95% CI)	RRR(95% CI)	RRR(95% CI)	RRR(95% CI)	RRR(95% CI)
**Sleep characteristics**								
**Sleep problem**								
No sleep problem	Ref	Ref					Ref	Ref
Sleep problem	0.96 (0.42–2.10)	6.90 (4.49–10.60)***					1.29 (0.52–3.21)	13.37 (7.14–25.05)***
**Obstructive sleep apnea (OSA)**								
No	Ref	Ref					Ref	Ref
Yes	1.31 (0.43–3.97)	3.02 (1.58–5.75)**					1.83 (0.52–6.44)	3.21 (1.31–7.86)*
**Adolescents dietary habit in past 30 days**								
**Went hungry**								
Never	Ref	Ref					Ref	Ref
Rarely/sometimes	2.70 (1.43–5.08)**	1.02 (0.66–1.58)					2.56 (1.27–5.16)**	0.86 (0.48–1.53)
**Consumed fruits**								
Never	Ref	Ref					Ref	Ref
≥ 1 times/day	2.01 (1.08–3.71)*	1.38 (0.90–2.09)					1.94 (0.98–3.83)	1.32 (0.78–2.26)
**Consumed vegetables**								
≤ 1 time/day	Ref	Ref					Ref	Ref
> 2 times/day	0.78 (0.39–1.54)	1.14 (0.71–1.84)					0.68 (0.31–1.48)	1.27 (0.68–2.36)
**Consumed soft drinks**								
< 1 time/day	Ref	Ref					Ref	Ref
≥ 1 times/day	0.27 (0.13–0.54)***	5.12 (3.13–8.36)***					0.27 (0.12–0.61)**	5.44 (2.93–10.10)***
**Consumed high fat food**								
≤ 1 time/day	Ref	Ref					Ref	Ref
≥ 2 times/day	1.01 (0.52–1.91)	2.23 (1.46–3.41)***					1.12 (0.48–2.65)	2.17 (1.18–3.99)*
**Consumed fast food**								
Daily	Ref	Ref					Ref	Ref
2–3 days	0.29 (0.13–0.66)**	1.06 (0.49–2.27)					0.62 (0.23–1.69)	1.08 (0.40–2.92)
≥ 4 days	0.23 (0.09–0.55)**	1.59 (0.74–3.44)					0.45 (0.15–1.31)	1.25 (0.46–3.39)
**Consumed junk food**								
No	Ref	Ref					Ref	Ref
Yes	1.95 (0.98–3.90)	3.79 (2.08–6.70)***					1.89 (0.87–4.09)	5.71 (2.55–12.82)***
**Consumed food groups (10 food groups)**								
< 5 food groups	2.86 (1.54–5.33)**	1.24 (0.81–1.90)					0.27 (0.13–0.57)**	0.55 (0.31–0.97)*
≥ 5 food groups	Ref	Ref					Ref	Ref
**Adolescents characteristics**								
**Age (years)**								
12–13			Ref	Ref			Ref	Ref
14–16			1.22 (0.51–2.91)	0.34 (0.22–0.52)***			1.26 (0.48–3.34)	0.29 (0.16–0.52)***
**Sex**								
Female								
Male			Ref	Ref			–	–
**Type of school**			1.05 (0.57–1.93)	0.96 (0.65–1.43)			–	–
Public								
Private			Ref	Ref			–	–
			0.69 (0.36–1.31)	1.50 (0.95–2.37)			–	–
**Parents characteristics**								
**Ethnicity**								
Brahmin/Chhetri			Ref	Ref			Ref	Ref
Terai Madhesi castes			1.37 (0.41–4.55)	1.33 (0.70–2.54)			1.32 (0.33–5.25)	2.81 (1.19–6.64)*
Dalit and minorities			6.20 (2.89–13.29)***	0.86 (0.43–1.71)			5.61 (2.12–14.82)***	1.13 (0.44–2.90)
Janajati			1.13 (0.51–2.49)	0.72 (0.45–1.13)			1.07 (0.41–2.74)	1.14 (0.56–2.34)
**Family type**								
Nuclear			Ref	Ref			–	–
Joint			1.17 (0.63–2.18)	1.15 (0.77–1.71)			–	–
**Mothers education**								
Below high school (≤ 10 class)			Ref	Ref			–	–
High class (10 + 2)			1.22 (0.55–2.70)	0.76 (0.44–1.31)			–	–
University education			1.49 (0.51–4.37)	0.75 (0.37–1.53)			–	
**Fathers education**								
Below high school (≤ 10 class)			Ref	Ref			–	–
High class (10 + 2)			1.95 (0.69–5.56)	0.69 (0.35–1.35)			–	–
University education			1.38 (0.44–4.27)	0.75 (0.37–1.52)			–	–
**Mothers occupation**								
Unemployed			Ref	Ref			–	–
Employed			0.87 (0.42–1.76)	0.92 (0.58–1.48)			–	–
**Fathers occupation**								
Unemployed			Ref	Ref			Ref	Ref
Employed			0.48 (0.21–1.09)	2.13 (1.15–3.93)*			1.03 (0.49–2.18)	2.04 (1.04–3.98)*
**Monthly income (NRs)(1USD = 110NRs)**								
< 15,000			Ref	Ref			–	–
15,000–30,000			0.63 (0.27–1.46)	0.69 (0.39–1.21)			–	–
30,000–45,000			1.32 (0.49–3.59)	0.74 (0.39–1.43)			–	–
> 45,000			0.91 (0.32–2.62)	0.71 (0.36–1.38)			–	–
**Adolescents physical activity**								
**Physical active**								
Not active					Ref	Ref	Ref	Ref
Active					0.35 (0.15–0.78)*	0.66 (0.38–0.95)*	0.34 (0.14–0.80)*	0.64 (0.36–1.12)
**Walking or ride bicycle**								
None					Ref	Ref	Ref	Ref
1–3 days/week					0.95 (0.39–2.34)	2.76 (1.53–4.95)**	0.81 (0.30–2.14)	4.33 (2.01–9.28)***
≥ 4 days/week					0.57 (0.25–1.30)	2.17 (1.31–3.61)**	0.66 (0.28–1.55)	1.86 (1.01–3.42)*
**Physical education/ training**								
None					Ref	Ref	Ref	Ref
1–3 days/week					0.27 (0.09–0.81)*	0.65 (0.37–1.14)	0.25 (0.08–0.80)*	0.50 (0.24–1.03)
≥ 4 days/week					0.40 (0.18–0.88)*	0.26 (0.14–0.48)***	0.48 (0.21–1.09)	0.24 (0.11–0.50)
**Sedentary behavior**								
< 1 h/day					Ref	Ref	Ref	Ref
1–2 h/day					0.51 (0.19–1.33)	0.77 (0.36–1.64)	0.43 (0.16–1.16)	0.86 (0.35–2.08)
3–4 h/day					0.40 (0.16–0.97)	0.84 (0.41–1.72)	0.30 (0.11–.0.80)*	0.69 (0.29–1.63)
5–6 h/day					0.69 (0.18–2.63)	5.71 (2.43–13.43)***	0.57 (0.14–2.31)	3.21 (1.14–9.09)*

Normal BMI: base outcome in multinomial logistic regression analysis

Ref: reference category for predictor variables

Model-I: adjusted for sleep characteristics and dietary habit

Model-II: adjusted for sleep characteristics, dietary habit and socio-demographic characteristics of parents and students

Model-III: adjusted for Physical activity and socio-demographic characteristics of parents and students and all variable shown under Model-III

Model- IV: adjusted for significant variables (*p* < 0.05) from and Model-I and Model-II, and students physical activity information and all variables shown under Model-IV

*RRR* relative risk ratio

* < 0.05, ** < 0.01, and *** < 0.001

After mutually adjusted all the confounding variables including socio-demographic, physical activity, dietary habits, and sleep characteristic, several factors showed a significantly higher risk of being overweight/obese: adolescents who had sleep problem compared to no sleep problem (RRR = 13.37, 95% CI: 7.14–25.05), adolescents who had OSA symptoms compared to no OSA symptoms (RRR = 3.21, 95% CI:1.31–7.86), who consumed soft drink ≥1 time/day compared to < 1 time/day in past 1 months (RRR = 5.44, 95% CI: 2.93–10.10), consumed high-fat food ≥2 times/day compared to ≤1 time/day (RRR = 2.17, 95% CI: 1.18–3.99), and adolescents who had consumed junk food compared to never consumed junk food (RRR = 5.71, 95% CI:2.55–12.82). Parents who belonged to Terai/Madhesi castes had more than two times (RRR = 2.81, 95% CI: 1.19–6.64) increased risk of overweight/obese children compared to Brahmin/Chhetri castes. Adolescents whose fathers were employed also had more than two times (RRR = 2.04, 95% CI: 1.04–3.98) significantly higher risk of being overweight/obese. Adolescents who used bicycle or walked 1–3 days/week as their primary means of transport to and from school or around the home (RRR = 4.33, 95% CI: 2.01–9.28), and ≥ 4 days/week (RRR = 1.86, 95% CI: 1.01–3.42) as their primary means of transport to and from school or around the home were significantly higher risk of being overweight/obesity compared to those who didn’t. Adolescents who had spent 5–6 h/day in sedentary behavior were 3.21 times (RRR = 3.21, 95% CI: 1.14–9.09) increased risk of being overweight/obese than those who had spent < 1 h/day in sedentary behavior. In contrast, adolescents aged 14–16 years were 71% lower (RRR = 0.29, 95% CI: 0.16–0.52) and adolescents who had consumed less than five food groups had 45% lower (RRR = 0.55, 95% CI: 0.31–0.97) risk of being overweight/obesity compared to 12–14 years age groups and who had consumed more than five food groups respectively.

The relative risk of underweight was found increased in adolescents who rarely/sometimes went hungry (RRR = 2.56, 95% CI: 1.27–5.16) compared to never went hungry, and Dalit and minority castes (RRR = 5.61, 95% CI: 2.12–14.82) compared to Brahmin/Chhetri castes. In contrast, those adolescents who consumed soft drink had 73% (RRR = 0.27, 95% CI: 0.12–0.61) than those who consumed < 1 time/day, physically active had 66% (RRR = 0.34, 95% CI: 0.14–0.80) compared to physically inactive, received 1–3 days physical education/training during this school year had 75% (RRR = 0.25, 95% CI: 0.08–0.80) compared to no physical education/training, and those who spent 3–4 h/ day in sedentary behavior had 43% (RRR = 0.57, 95% CI: 0.11–0.80) compared to those who spent < 1 h/day in sedentary behavior respectively were at lower risk of being underweight (Table 3).

## Discussion

To the best of our knowledge, this is one of the first studies that examine the association of sleep with body mass index among Nepalese adolescents. Our study revealed that one-fifth of the adolescents (23.7%) were found to be overweight/obese, which is consistent with results from the previous studies conducted in Nepal (25–26% overweight/obesity) [11, 12]. Most independent variables showed a significant association with overweight/obesity after mutual adjustment of confounding variables, including socio-demographic, physical activity, dietary habits and sleep characteristics.

### Sleep problem and weight status

We found nearly one in four adolescents (24.4%) had sleep problems and similar findings have been reported from several studies conducted in both developed and developing countries across the globe that showed sleep disorders as a common problem, particularly among the teenage population [53–56]. In this study, adolescents’ sleep duration during weekdays and weekends, OSA symptoms, and sleep problems measured by CASC were significantly different among different groups for the BMI for the age of the adolescents. Also, adolescents who had sleep problems were found more likely to be overweight/obese. These findings are similar to the evidence from several previous studies conducted in different geographical regions of the world, where they found increased risks of overweight/obesity among young age groups of participants are those having sleep problems or short sleep duration [25, 57]. Several factors are responsible for the association of sleep problems and overweight/obesity, including both biological and habitual. The biological phenomenon can be explained with the evidence that sleep deprivation is associated with dysregulation of neuroendocrine control of appetites such as alterations in the hormones, including cortisol, leptin, and ghrelin [58, 59]. These hormonal changes are further responsible for alterations in demand for body calorie requirements. As a result, individuals adopt unhealthy food choices, consume additional foods, and decrease their physical activities.

Likewise, about one in every ten adolescents were found to have OSA symptoms, and those who had OSA symptoms were found to be at higher risks of overweight/obesity compared to those who did not have OSA symptoms. There is ample of evidence that suggests vice-versa associations of OSA and overweight/obesity among children and adolescents. The study conducted among Australian adolescents showed that with each standard deviation increase in BMI z-score, there is more than three times more risk of developing OSA, which supports the findings of our study [60]. Mitchell and Kelly [61] study claim that one in two obese children had OSA in the United States of America (USA). Severe upper ways obstruction in obese children can lead to sleep disorders further resulting in causing obesity and risk factors for cardiovascular disease, stroke, metabolic disease, and day-time sleepiness [62].

### Dietary habits and weight status

Due to “nutrition transition” in most of the low-income countries the food consumption patterns has shifted from locally available staple foods consumption towards the consumption of western diet pattern containing poor nutrients, foods high in fat, energy-dense foods, sugar-sweetened beverages, junk foods, fast foods and less consumption of vegetables and fruits [63]. In this study, adolescents who consumed soft drinks ≥ one time per day and high-fat food ≥ two times per day in the last 30 days and who consumed junk food were found to have higher risks of developing overweight/obesity. A recent review study shows that Asian pre-school children adhering to the westernized eating pattern, which is described as a high intake of sugary beverages, snacks, and red meat was found to have significantly higher odds of being obese [64]. Likewise, Brazilian children adhering to junk food diets [64], Chinese adolescents who followed the Western dietary patterns [64], Indian and Saudi adolescents who practiced adverse eating behaviors including consumption of high-fat foods and carbonate drinks were also found significantly at higher risk of being obese [65–67]. However, the review by Togo et al. [68] presents mixed findings from 30 different studies where few studies showed negative or no association of high fatty, sweets, or energy-dense food consumption habits with overweight/obesity. The difference in the pattern of results might be due to the complexity of determining factors for overweight/obesity [31] and also, in fact, eating pattern alone is not only responsible for producing overweight/obesity.

Our study also revealed that adolescents who had consumed < five food groups within the last 24 h preceding to survey were found protective towards overweight/obesity as compared to those who consumed ≥ five food groups. These findings are supported by the results of the study conducted in the USA, where diversified food group consumption was positively associated with body fatness [69]. However, the systematic review by Otto et al. [70] shows that there is inconsistency in the results regarding the influence of dietary diversity on overweight/obesity [70].

On the other hand, only a few food-related factors were associated with an increased risk of underweight. The relative risk of underweight was found increased in adolescents who rarely/sometimes went hungry. Similar findings are reported by Johnson et al. [71], where they reported that going hungry most of the time was marginally associated with an increased risk of underweight [71]. Those adolescents who consumed soft drinks had a 73% lower risk of underweight than those who consumed <one time/day. This finding is in line with the previous study, where those adolescents who drank soft drinks had a 17% lower risk of underweight [63]. Food containing high sugar-sweetened beverages is linked to increased body weight [63]. In this study, adolescents who had consumed less than five food groups had a 77% lower risk of being underweight. Household food insecurity and lack of access to diversified food households were likely to suffer malnutrition because of very limited intake of nourishing food resulting in underweight adolescents. The decline in their fewer food groups was primarily due to lower consumption of meat and poultry, fish and shrimp, eggs, and dairy products [72].

### Physical activity and weight status

Our study revealed that very low percentages of adolescents were found to be involved in physical activity and more than half of the adolescents were found to have more than 3 h of sedentary lifestyle per day. These results were found congruent with the findings from similar studies conducted in the past [48, 73]. However, these results are explicitly indicating that some of these modifiable risk factors linked to obesity presented among Nepalese adolescents, if not changed at the early stages of life, may continue to adulthood and exacerbate the burden of chronic illness in the future. Also, our study presents those who spend 5–6 h of sedentary lifestyle per day were more than three times higher risk of overweight/obesity compared to those who spend less than one-hour sedentary lifestyle per day. On the other hand, those who attained physical education or training ≥4 days/week were less likely to be overweight/obese when compared to those who do not attain any physical education or training in the recent week in this school year. It is more likely that those who were involved in insufficient physical activity and spent less sedentary time had a positive influence on maintaining normal BMI [74]. It was also found that those who were involved in walking or riding a bicycle ≥4 days/week were slightly more at risk of overweight/obesity, which sounds quite unexpected. This may be due to the reason that adolescents who were overweight/obese might have recently engaged in walking or riding a bicycle as a form of physical activity to reduce their weight. Another reason may be due to certain limitations such as behavioral findings were assessed using a self-administered questionnaire. Thus, social desirability bias leading to over and under-reporting may occur, and tools used in physical activity were taken only for the past 1 week.

### Socio-demographic characteristic and weight status

In the current study, adolescents belonging to Terai/Madhesi castes were found more likely to be overweight/obese. There is a paucity of evidence demonstrating the association between ethnicity and overweight/obesity among adolescents; as a result, we couldn’t justify it anymore in this study. These findings recommend prospective researchers to explore the causal relationship between ethnic variations and BMI within the Nepalese context. However, the possible reason could be that the elite group or richest people in Nepal comes from Madhesi, Newar, Thakali, and Thakuri ethnic groups, alongside Bhramin and Chhetri. Those richest people who belonged to Terai/Madhesi caste were migrated from Terai to Kathmandu and are privileged to study in high-quality English-medium schools. Moreover, the richest peoples have access to energy-dense, fast foods in their family, and luxurious lifestyle and able to high daily expenses resulting in overweight/obese children. In contrast, adolescents who belonged to Dalit and minority caste were found to be underweight. Dalit castes, one of the most marginalized groups in Nepal, are still facing several forms of discrimination in all sectors, including the workplace. As a result, Dalits today stand at the bottom of most indicators of socio-economic development [75]. Peoples from low-income families lack access to resources to purchase healthy and sufficient foods for their families, resulting in their children being underweight [9]. Despite the formulation of several plans, policies, and legislation to abolish caste-based discrimination in Nepal, the disadvantaged ethnic groups are facing different forms of discrimination [76]. To uplift, the socio-economic status of such disadvantaged ethnic groups warrant effective implementation of existing governmental plans, policies, and legislation. Prevention of modifiable risk factors such as poor dietary habits, physical inactivity, sedentary behavior, and sleep deficiency for both under and over-nutrition among adolescents can be achieved through the implementation of nutrition-specific and nutrition-sensitive interventions [77, 78]. Our findings suggest that the father’s employment status was found to have increased the risk of overweight/obese children. This finding is in line with the other study, where they found that father’s employment to be associated with overweight/ obese childhood [79]. The possible reason could be those adolescents whose fathers were in employment have higher socio-economic income sources and access to purchasing more calorie-dense and nutrient-poor foods. Those adolescents who were between aged 14–16 years were less likely to be overweight/obese as compared to those who were between aged 12–13 years. These findings are consistent with Smetanina et al. [80] results, where they reported that overweight/obesity is decreased with an increase in age. The plausible reason could be that adolescents are more likely to increase the physical exercise in school/home, and are self-aware about their health status with an increase in their age.

### Limitation and strength

Our study has some limitations. First, due to the cross-sectional study design of the study, the directional cause and effect relationship between independent and dependent variables cannot be inferred. Generalizing the pattern of dietary habits, sleep characteristics, and physical activity throughout the entire calendar year would be misleading as the data were collected at a specific point of time. Second, the data collected were self-reported and it was retrospective, we could not completely ignore the recall bias. Third, calculating the actual amount of energy expenditure or sedentary behavior was not possible due to the use of self-reported information. Likewise, food consumption in terms of the nutrient amount was not calculated, which could result in overestimating or underestimating the actual extent of dietary habits influences on the BMI of adolescents. Fourth, since the participants were recruited from the schools located in urban settings, the results of this study cannot be generalized among adolescents living in rural areas of the country. Despite the mentioned limitations, our study has some strengths. This study is the first of its kind to explore the association of sleep characteristics with BMI of adolescents in the Nepalese context. Therefore, this study fills the gap of evidence regarding how sleep characteristics with dietary habits and physical activity influence the BMI status of Nepalese adolescents. Further, this study has attempted to bring the attention of researchers for the future in-depth study for the explicit understanding of the multifactor and the complexity of the overweight/obesity mechanism among Nepalese adolescents, which is one of the major current public health concerns in the country.

## Conclusions

The current study highlighted that overweight/obesity among Nepalese adolescents is at an alarming level and on par with many developing and developed countries. Sleep problem, OSA, consumption of carbonated /soft drinks, consumptions of fast foods and junk foods, physical education/training, sedentary behaviors, participant’s age, ethnicity, and father occupation status were major predictors for overweight/obesity among adolescents. The findings of this study warrant immediate interventions to improve lifestyle behavior and reduce overweight/obesity among adolescents. Creating a conducive environment both at school and home to encourage adolescents to adopt a healthy lifestyle and dietary choices are crucial in reducing overweight/obesity. It is also essential to regularly monitor the BMI status, sleep quality, and health behaviors of adolescents so that it would be possible to modify their dietary habits and other lifestyle behaviors timely. Adolescents and their parents need to be aware of the minimum sleep duration required for adolescents and also about the impact of inadequate sleeping on health, which could help to improve the sleep quality of adolescents. Finally, the concerned authorities are required to develop long-term practical health-promoting policies to reduce the risk of overweight/obesity as well as to improve the overall wellbeing of Nepalese adolescents.

## Data Availability

All data analyzed during this study are available from the corresponding author on reasonable request.

## Abbreviations

BMI: Body Mass Index
CSAC: Child and Adolescents Sleep Checklist
NCDs: Non-communicable Diseases
OSA: Obstructive Sleep Apnea
OW/OB: Overweight/obesity
WHO: World Health Organization

## References

[1] Chaput JP, Dutil C (2016). Lack of sleep as a contributor to obesity in adolescents: impacts on eating and activity behaviors. Int J Behav Nutr Phys Act.

[2] Gluckman P, Nishtar S, Armstrong T (2015). Ending childhood obesity: a multidimensional challenge. Lancet.

[3] World Health Organization (2016). Obesity and overweight: key facts.

[4] Bentham J, Di Cesare M, Bilano V, Bixby H, Zhou B, Stevens GA (2017). Worldwide trends in body-mass index, underweight, overweight, and obesity from 1975 to 2016: a pooled analysis of 2416 population-based measurement studies in 128·9 million children, adolescents, and adults. Lancet..

[5] Lobstein T, Brinsden H (2019). World obesity : atlas of Chidhood obesity.

[6] Bhurosy T, Jeewon R (2014). Overweight and obesity epidemic in developing countries: a problem with diet, physical activity, or socioeconomic status?. Sci World J.

[7] World Health Organization (2016). Commission on Ending Childhood : Facts and figures on childhood obesity.

[8] Mistry SK, Puthussery S (2015). Risk factors of overweight and obesity in childhood and adolescence in south Asian countries: a systematic review of the evidence. Public Health.

[9] Bhattarai S, Kant BC (2019). Prevalence and associated factors of malnutrition among school going adolescents of dang district, Nepal. AIMS Public Heal.

[10] Hasan M, Sutradhar I, Shahabuddin A, Sarker M. Double burden of malnutrition among Bangladeshi women: a literature review. Cureus. 2017;9. 10.7759/cureus.1986. 10.7759/cureus.1986 PMC5826745 29503780

[11] Karki A, Shrestha A, Subedi N (2019). Prevalence and associated factors of childhood overweight/obesity among primary school children in urban Nepal. BMC Public Health.

[12] Koirala M, Khatri RB, Khanal V, Amatya A (2015). Prevalence and factors associated with childhood overweight/obesity of private school children in Nepal. Obes Res Clin Pract.

[13] Kumar Aryal K, Bista B, Bahadur Khadka B, Raj Pandey A, Mehta R, Kumar Jha B (2017). Global School Based Student Health Survey Nepal, 2015.

[14] Verstraeten R, Leroy JL, Pieniak Z, Ochoa-Avilès A, Holdsworth M, Verbeke W, Maes L, Kolsteren P (2016). Individual and environmental factors influencing adolescents’ dietary behavior in low- and middle-income settings. PLoS One.

[15] Gupta N, Goel K, Shah P, Misra A (2012). Childhood obesity in developing countries: epidemiology, determinants, and prevention.

[16] Narciso J, Silva AJ, Rodrigues V, Monteiro MJ, Almeida A, Saavedra R, Costa AM (2019). Behavioral, contextual and biological factors associated with obesity during adolescence: a systematic review. PLoS One.

[17] Lytle LA (2009). Examining the etiology of childhood obesity: the idea study. Am J Community Psychol.

[18] Ohri-Vachaspati P, DeLia D, DeWeese RS, Crespo NC, Todd M, Yedidia MJ (2015). The relative contribution of layers of the social ecological model to childhood obesity. Public Health Nutr.

[19] World Health Organization. Global status report on non-communicable diseases 2014. Geneva; 2014. https://apps.who.int/iris/bitstream/handle/10665/148114/9789241564854_eng.pdf?sequence=1. Accessed 16 May 2020

[20] Labayen Goñi I, Arenaza L, Medrano M, García N, Cadenas-Sanchez C, Ortega FB (2018). Associations between the adherence to the Mediterranean diet and cardiorespiratory fitness with total and central obesity in preschool children: the PREFIT project. Eur J Nutr.

[21] Love R (2005). A good night’s sleep is the key to remembering. Lancet Neurol.

[22] Otte A, Turkheimer F, Rosenzweig I (2016). All you need is sleep. EBioMedicine..

[23] Dahl RE, Lewin DS (2002). Pathways to adolescent health: sleep regulation and behavior. J Adolesc Health.

[24] Hirshkowitz M, Whiton K, Albert SM, Alessi C, Bruni O, DonCarlos L, Hazen N, Herman J, Adams Hillard PJ, Katz ES, Kheirandish-Gozal L, Neubauer DN, O’Donnell AE, Ohayon M, Peever J, Rawding R, Sachdeva RC, Setters B, Vitiello MV, Ware JC (2015). National Sleep Foundation’s updated sleep duration recommendations: final report. Sleep Heal.

[25] Gohil A, Hannon TS (2018). Poor sleep and obesity: concurrent epidemics in adolescent youth. Front Endocrinol (Lausanne).

[26] Medic G, Wille M, Hemels MEH (2017). Short- and long-term health consequences of sleep disruption. Nat Sci Sleep.

[27] Ayas NT, White DP, Manson JAE, Stampfer MJ, Speizer FE, Malhotra A, Hu FB (2003). A prospective study of sleep duration and coronary heart disease in women. Arch Intern Med.

[28] Fredriksen K, Rhodes J, Reddy R, Way N (2004). Sleepless in Chicago: tracking the effects of adolescent sleep loss during the middle school years. Child Dev.

[29] Bjorvatn B, Sagen IM, Øyane N, Waage S, Fetveit A, Pallesen S (2007). The association between sleep duration, body mass index and metabolic measures in the Hordaland Health Study. J Sleep Res.

[30] Al-Kloub MI, Froelicher ES (2009). Factors contributing to adolescent obesity. Saudi Med J.

[31] Hruby A, Hu FB (2015). The epidemiology of obesity: a big picture. PharmacoEconomics..

[32] Raychaudhuri M, Debmalya S. Childhood obesity: determinants, evaluation, and prevention. Indian J Endocrinol Metab. 2012;6 https://www.ncbi.nlm.nih.gov/pmc/articles/PMC3603024/. Accessed 5 Dec 2020. 10.4103/2230-8210.104037 PMC3603024 23565376

[33] Popkin BM, Adair LS, Ng SW (2012). Global nutrition transition and the pandemic of obesity in developing countries. Nutr Rev.

[34] Prasad Subedi YM, Marais D, Newlands DM (2017). Where is Nepal in the nutrition transition?. Asia Pac J Clin Nutr.

[35] Central Bureau of Statistics. Population Monograph of Nepal (Volume-I) : Population Dynamics. Kathmandu: Central Bureau of Statistics, National Planning Commission, Government of Nepal; 2014. https://nepal.unfpa.org/sites/default/files/pub-pdf/PopulationMonograph2014Volume1.pdf. Accessed 10 Dec 2020

[36] Vaidya A, Shakya S, Krettek A (2010). Obesity prevalence in Nepal: public health challenges in a low-income nation during an alarming worldwide trend. Int J Environ Res Public Health.

[37] Dhungana RR, Bista B, Pandey AR, De Courten M (2019). Prevalence, clustering and sociodemographic distributions of non-communicable disease risk factors in nepalese adolescents: secondary analysis of a nationwide school survey. BMJ Open.

[38] Central Bureau of Statistics. National Population and Housing Census 2011. Kathmandu; 2012. https://unstats.un.org/unsd/demographic/sources/census/wphc/Nepal/Nepal-Census-2011-Vol1.pdf. Accessed 21 July 2018

[39] Arya R, Antonisamy B, Kumar S (2012). Sample size estimation in prevalence studies. Indian J Pediatr.

[40] KMC (2019). Kathmandu Metropolitan City | Office of Municipal Executive, Bagmati Province.

[41] World Health Organization (2007). Growth reference 5–19 years: BMI-for-age (5–19 years).

[42] Oka Y, Horiuchi F, Tanigawa T, Suzuki S, Kondo F, Sakurai S, Saito I, Tanimukai S (2009). S Ueno YI. Development of a new sleep screening questionnaire: child and adolescent sleep checklist (CASC). Japanese J Sleep Med.

[43] Tabitha Louis P, Arnold EI (2014). Evaluating the cognition, behavior, and social profile of an adolescent with learning disabilities and assessing the effectiveness of an individualized educational program. Iran J psychiatry Behav Sci.

[44] Sivertsen B, Øverland S, Glozier N, Bjorvatn B, Mæland JG, Mykletun A (2008). The effect of OSAS on sick leave and work disability. Eur Respir J.

[45] Sivertsen B, Pallesen S, Sand L, Hysing M (2014). Sleep and body mass index in adolescence: results from a large population-based study of Norwegian adolescents aged 16 to 19 years. BMC Pediatr.

[46] WHO. Global School-Based Students Health Survey (GSHS): 2015 Nepal GSHS Questionnaire. Geneva; 2015. https://www.who.int/ncds/surveillance/gshs/2015_GSHS_Nepal_Questionnaire-Nepali.pdf. Accessed 1 May 2020

[47] FAO and FANTA. Minimum dietary diversity for women:a guide to measurement. Rome; 2016. http://www.fao.org/3/a-i5486e.pdf. Accessed 9 Sep 2019

[48] Kumar Aryal K, Bista B, Bahadur Khadka B, Raj Pandey A, Mehta R, Kumar Jha B (2015). Global school based student health survey Nepal-2015.

[49] Guthold R, Stevens GA, Riley LM, Bull FC (2019). Global trends in insufficient physical activity among adolescents: a pooled analysis of 298 population-based surveys with 1Â·6 million participants. Artic Lancet Child Adolesc Heal.

[50] WHO. Global recommendations on physical activity for health. Geneva; 2011. https://www.who.int/dietphysicalactivity/publications/physical-activity-recommendations-5-17years.pdf?ua=1. Accessed 2 May 2020

[51] Bennett L, Ram Dahal D, Govindasamy P. Caste, Ethnic and Regional Identity in Nepal : Further Analysis of the 2006 Nepal Demographic and Health Survey. Calverton, Maryland, USA; 2008. https://dhsprogram.com/pubs/pdf/FA58/FA58.pdf.

[52] Midi H, Sarkar SK, Rana S (2010). Collinearity diagnostics of binary logistic regression model. J Interdiscip Math.

[53] Mathew G, Varghese AD, Benjamin AI (2019). A comparative study assessing sleep duration and associated factors among adolescents studying in different types of schools in an urban area of Kerala, India. Indian J Community Med.

[54] Guo L, Deng J, He Y, Deng X, Huang J, Huang G, Gao X, Lu C (2014). Prevalence and correlates of sleep disturbance and depressive symptoms among Chinese adolescents: a cross-sectional survey study. BMJ Open.

[55] Kidwai R, Ahmed SH (2013). Prevalence of insomnia and use of sleep medicines in urban communities of Karachi, Pakistan. J Pak Med Assoc.

[56] Kotagal S, Pianosi P (2006). Sleep disorders in children and adolescents. BMJ..

[57] Patel SR, Hu FB (2008). Short sleep duration and weight gain: a systematic review. Obesity..

[58] Felső R, Lohner S, Hollódy K, Erhardt E, Molnár D (2017). Relationship between sleep duration and childhood obesity: systematic review including the potential underlying mechanisms. Nutr Metab Cardiovasc Dis.

[59] Chen X, Beydoun MA, Wang Y, Lomeli HA, Perez-Olmos I, Talero-Gutierrez C (2008). Is sleep duration associated with childhood obesity? A systematic review and meta-analysis\nSleep evaluation scales and questionaries: a review. Obesity (Silver Spring).

[60] Kohler MJ, Thormaehlen S, Kennedy JD, Pamula Y, Van Den Heuvel CJ, Lushington K (2009). Differences in the association between obesity and obstructive sleep apnea among children and adolescents. J Clin Sleep Med.

[61] Mitchell RB, Kelly J (2004). Adenotonsillectomy for obstructive sleep apnea in obese children. Otolaryngol - Head Neck Surg.

[62] Knauert M, Naik S, Gillespie MB, Kryger M (2015). Clinical consequences and economic costs of untreated obstructive sleep apnea syndrome. World J Otorhinolaryngol Neck Surg.

[63] Popkin BM (1998). The nutrition transition and its health implications in lower-income countries. Public Health Nutr.

[64] Liberali R, Kupek E, Altenburg De Assis MA (2020). Dietary patterns and childhood obesity risk: a systematic review.

[65] Faizi N, Shah M, Ahmad A, Ansari M, Amir A, Khalique N (2018). Adverse eating behavior and its association with obesity in Indian adolescents: evidence from a nonmetropolitan city in India. J Fam Med Prim Care.

[66] Amin T, Al-Sultan A, Ali A (2008). Overweight and obesity and their association with dietary habits, and sociodemographic characteristics among male primary school children in Al-Hassa, Kingdom of Saudi Arabia. Indian J Community Med.

[67] Joseph N, Nelliyanil M, Rai SYPRB, Kotian SM, Ghosh T (2015). Fast food consumption pattern and its association with overweight among high school boys in Mangalore City of Southern India. J Clin Diagnostic Res.

[68] Togo P, Osler M, Sørensen TIA, Heitmann BL (2001). Food intake patterns and body mass index in observational studies. Int J Obes.

[69] McCrory MA, Fuss PJ, McCallum JE, Yao M, Vinken AG, Hays NP (1999). Dietary variety within food groups: association with energy intake and body fatness in men and women. Am J Clin Nutr.

[70] Otto C de OM, Anderson CAM, Dearborn JL, Ferranti EP, Mozaffarian D, Rao G, et al. Dietary Diversity: Implications for Obesity Prevention in Adult Populations: A Science Advisory From the American Heart Association. Circulation. 2018;138:e160–8. 10.1161/CIR.0000000000000595. 10.1161/CIR.0000000000000595 PMC6894732 30354383

[71] Johnson RK, Lamb M, Anderson H, Pieters-Arroyo M, Anderson BT, Bolaños GA, et al. The global school-based student health survey as a tool to guide adolescent health interventions in rural Guatemala. BMC Public Health. 2019;19:1–9. 10.1186/s12889-019-6539-1. 10.1186/s12889-019-6539-1 PMC6387528 30795754

[72] Yang Q, Yuan T, Yang L, Zou J, Ji M, Zhang Y, et al. Household food insecurity, dietary diversity, stunting, and anaemia among left-behind children in poor rural areas of China. Int J Environ Res Public Health. 2019;16(23). 10.3390/ijerph16234778. 10.3390/ijerph16234778 PMC6926723 31795269

[73] Thapa K, Bhandari PM, Neupane D, Bhochhibhoya S, Rajbhandari-Thapa J, Pathak RP (2019). Physical activity and its correlates among higher secondary school students in an urban district of Nepal. BMC Public Health.

[74] McManus AM, Mellecker RR (2012). Physical activity and obese children. J Sport Health Sci.

[75] International Labour Organization. Dalits and Labour in Nepal: Discrimination and Forced Labour. Kathamndu , Nepal; 2005. https://www.ilo.org/wcmsp5/groups/public/@asia/@ro-bangkok/@ilo-kathmandu/documents/publication/wcms_112922.pdf.

[76] Pariyar B, Lovett JC (2016). Dalit identity in urban Pokhara, Nepal. Geoforum.

[77] Ruel MT, Alderman H (2013). Nutrition-sensitive interventions and programmes: how can they help to accelerate progress in improving maternal and child nutrition?. Lancet.

[78] Chaput JP, Tremblay A (2012). Adequate sleep to improve the treatment of obesity. CMAJ..

[79] Hope S, Pearce A, Whitehead M, Law C (2015). Parental employment during early childhood and overweight at 7-years: findings from the UK Millennium Cohort Study.

[80] Smetanina N, Albaviciute E, Babinska V, Karinauskiene L, Albertsson-Wikland K, Petrauskiene A, et al. Prevalence of overweight/obesity in relation to dietary habits and lifestyle among 7-17 years old children and adolescents in Lithuania health behavior, health promotion and society. BMC Public Health. 2015;15:1–9. 10.1186/s12889-015-2340-y. 10.1186/s12889-015-2340-y PMC4590263 26429124

